# Cryo-EM and biochemical analyses of the nucleosome containing the human histone H3 variant H3.8

**DOI:** 10.1093/jb/mvad069

**Published:** 2023-09-26

**Authors:** Seiya Hirai, Tomoya Kujirai, Munetaka Akatsu, Mitsuo Ogasawara, Haruhiko Ehara, Shun-ichi Sekine, Yasuyuki Ohkawa, Yoshimasa Takizawa, Hitoshi Kurumizaka

**Affiliations:** Laboratory of Chromatin Structure and Function, Institute for Quantitative Biosciences, The University of Tokyo, 1-1-1 Yayoi, Bunkyo-ku, Tokyo 113-0032, Japan; Department of Biological Sciences, Graduate School of Science, The University of Tokyo, 1-1-1 Yayoi, Bunkyo-ku, Tokyo 113-0032, Japan; Laboratory of Chromatin Structure and Function, Institute for Quantitative Biosciences, The University of Tokyo, 1-1-1 Yayoi, Bunkyo-ku, Tokyo 113-0032, Japan; Laboratory of Chromatin Structure and Function, Institute for Quantitative Biosciences, The University of Tokyo, 1-1-1 Yayoi, Bunkyo-ku, Tokyo 113-0032, Japan; Department of Biological Sciences, Graduate School of Science, The University of Tokyo, 1-1-1 Yayoi, Bunkyo-ku, Tokyo 113-0032, Japan; Laboratory of Chromatin Structure and Function, Institute for Quantitative Biosciences, The University of Tokyo, 1-1-1 Yayoi, Bunkyo-ku, Tokyo 113-0032, Japan; Laboratory for Transcription Structural Biology, RIKEN Center for Biosystems Dynamics Research, 1-7-22 Suehiro-cho, Tsurumi-ku, Yokohama 230-0045, Japan; Laboratory for Transcription Structural Biology, RIKEN Center for Biosystems Dynamics Research, 1-7-22 Suehiro-cho, Tsurumi-ku, Yokohama 230-0045, Japan; Division of Transcriptomics, Medical Institute of Bioregulation, Kyushu University, 3-1-1 Maidashi, Higashi, Fukuoka 812-0054, Japan; Laboratory of Chromatin Structure and Function, Institute for Quantitative Biosciences, The University of Tokyo, 1-1-1 Yayoi, Bunkyo-ku, Tokyo 113-0032, Japan; Laboratory of Chromatin Structure and Function, Institute for Quantitative Biosciences, The University of Tokyo, 1-1-1 Yayoi, Bunkyo-ku, Tokyo 113-0032, Japan; Department of Biological Sciences, Graduate School of Science, The University of Tokyo, 1-1-1 Yayoi, Bunkyo-ku, Tokyo 113-0032, Japan; Laboratory for Transcription Structural Biology, RIKEN Center for Biosystems Dynamics Research, 1-7-22 Suehiro-cho, Tsurumi-ku, Yokohama 230-0045, Japan

**Keywords:** chromatin, epigenetics, H3.8, histone variant, nucleosome

## Abstract

Histone H3.8 is a non-allelic human histone H3 variant derived from H3.3. H3.8 reportedly forms an unstable nucleosome, but its structure and biochemical characteristics have not been revealed yet. In the present study, we reconstituted the nucleosome containing H3.8. Consistent with previous results, the H3.8 nucleosome is thermally unstable as compared to the H3.3 nucleosome. The entry/exit DNA regions of the H3.8 nucleosome are more accessible to micrococcal nuclease than those of the H3.3 nucleosome. Nucleosome transcription assays revealed that the RNA polymerase II (RNAPII) pausing around the superhelical location (SHL) −1 position, which is about 60 base pairs from the nucleosomal DNA entry site, is drastically alleviated. On the other hand, the RNAPII pausing around the SHL(−5) position, which is about 20 base pairs from the nucleosomal DNA entry site, is substantially increased. The cryo-electron microscopy structure of the H3.8 nucleosome explains the mechanisms of the enhanced accessibility of the entry/exit DNA regions, reduced thermal stability and altered RNAPII transcription profile.

## Graphical Abstract

**Graphical Abstract:**
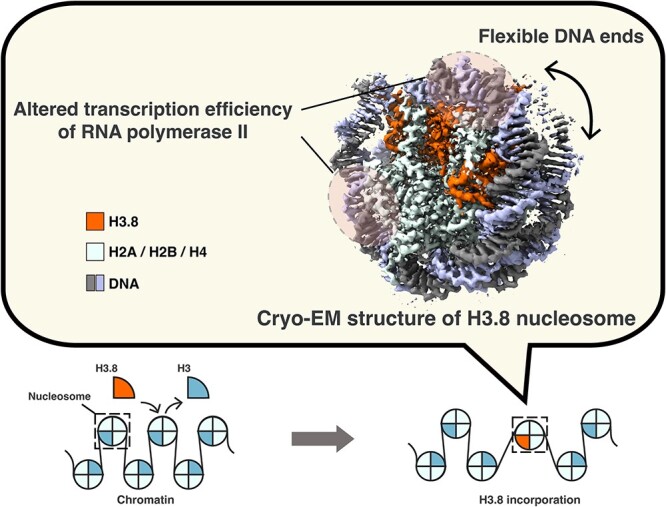

## Abbreviations

bp: base pair
CBB: Coomassie Brilliant Blue
CENP-A: centromere protein A
cryo-EM: cryo-electron microscopy
CTF: contrast transfer function
DTT: dithiothreitol
EDTA: ethylenediaminetetraacetic acid
EtBr: ethidium bromide
FSC: Fourier shell correlation
GraFix: gradient fixation
HEPES: 4-(2-hydroxyethyl)-1-piperazine ethanesulfonic acid
MNase: micrococcal nuclease
PAGE: polyacrylamide gel electrophoresis
RNAPII: RNA polymerase II
SDS: sodium dodecyl sulfate
SHLs: superhelical locations
TBE: tris-borate-EDTA
TFIIS: transcription elongation factor IIS

In eukaryotic cells, the fundamental structural unit of chromatin is the nucleosome, which is composed of histones H2A, H2B, H3 and H4, with 145 to 147 base pairs (bp) of DNA *(**1**)*. The core histones are categorized into canonical and variant types *(**2**)*. Canonical histones are encoded by multiple genes and produced during DNA replication in the S-phase of the cell cycle *(**3**)*. In contrast, histone variants are produced from a smaller set of genes, and their chromatin incorporation is not restricted to a particular phase of the cell cycle *(**2**,*  *4**)*. Histone variants are considered to have specialized functions in the regulation and maintenance of genomic DNA *(**2**)*.

Among the core histones, histone H3 has a diverse set of variants *(**2**,*  *5*–*8**)*. CENP-A is the centromere-specific H3 variant and forms a nucleosome with more accessible DNA ends as compared to the nucleosomes containing canonical H3.1 and the major variant H3.3 *(**9*–*12**)*. Enhanced nucleosomal DNA end accessibility is also evident in nucleosomes containing human H3.Y and mouse H3t *(**13**,*  *14**)*. Nucleosomes containing the human testis-specific histone variants, H3T and H3.5, are relatively unstable *(**15**,*  *16**)*. These nucleosome characteristics conferred by histone H3 variants may play important roles in genome regulation by chromatin.

Previously, we identified three novel human histone H3 variants, H3.6, H3.7 and H3.8 *(**17**,*  *18**)*. H3.6 and H3.8 retain the H3.3-specific amino acid residues, which are required for binding to the histone chaperones HIRA and DAXX *(**19*–*21**)*. In contrast, H3.7 appears to have evolved from H3.1 *(**17**,*  *18**)*. The H3.6 and H3.8 nucleosomes exhibit low stability. The crystal structure of the H3.6 nucleosome implied that the H3.6-specific Val62 residue may diminish the hydrophobic interactions with the cognate H4 molecule, as compared to the corresponding H3.3 Ile62 residue. However, the mechanism by which H3.8 induces nucleosome instability has remained enigmatic, because the structure of the H3.8 nucleosome has not been determined yet.

The human H3.8 gene is expressed in the ovary, adrenal glands, colon, kidney and thyroid *(**18**)*. In the present study, we biochemically characterized the H3.8 nucleosome and determined its structure by cryo-electron microscopy (cryo-EM) single particle analysis.

## Materials and Methods

### Preparation of human histones, histone complexes and nucleosomes

Human histone proteins, H2A, H2B, H3.3, H3.8 and H4, were bacterially produced and purified as described previously *(**22**)*. The H2A–H2B and H3–H4 complexes were prepared, and nucleosomes containing a 145-bp Widom 601L DNA or a 198-bp DNA for transcription were reconstituted and purified as described previously *(**22**,*  *23**)*. For the 145-bp Widom 601L DNA fragment, the 71-bp Widom 601L DNA with a 3-base 5′ overhang was self-ligated.

### Thermal stability assay of nucleosomes

The thermal stability of the nucleosome containing H3.3 or H3.8 was measured by a thermal stability assay, as described previously *(**24**)*. The nucleosome (24 pmol) was mixed with 5 × SYPRO Orange dye (Sigma-Aldrich) in 20 μl of reaction mixture (17 mM Tris–HCl buffer (pH 7.5), 0.9 mM dithiothreitol, 4.3% glycerol and 100 mM NaCl). A 19-μl portion of the mixture was subjected to a temperature gradient from 26 to 95°C, in steps of 1°C/min, and the fluorescence of SYPRO Orange was detected using a StepOnePlus™ Real-Time PCR system (Applied Biosystems). The fluorescent signals were normalized with the following formula: *F*(*T*)_normalized_ = [*F*(*T*) − *F*(26)]/[*F*(95) − *F*(26)], where *F*(*T*) indicates the fluorescence signal intensity at a particular temperature.

### Micrococcal nuclease assay

The nucleosomes (1.4 μg for DNA) containing the 145-bp DNA were incubated with 0.7 units of micrococcal nuclease (MNase) at 37°C in 70 μl of reaction solution (50 mM Tris–HCl (pH 7.5–8.0), 1.9 mM dithiothreitol, 2.5% glycerol, 25 mM NaCl and 2.5 mM CaCl_2_) for 0, 3, 6, 9, 12 and 15 min. Aliquots (10 μl) were taken at the indicated times and mixed with 5 μl of deproteinization solution (20 mM Tris–HCl (pH 8.0), 20 mM EDTA, 0.1% SDS and 0.49 mg/ml proteinase K (Roche)). The resulting reaction products were analyzed by non-denaturing 8% PAGE in 0.5× TBE buffer (45 mM Tris base, 45 mM boric acid and 1 mM EDTA), and the DNA fragments were visualized by ethidium bromide staining. The gel images were captured using an Amersham Imager 680 (Cytiva).

### RNA polymerase II transcription assay

RNA polymerase II (RNAPII), consisting of 12 subunits, was purified from a strain of *Komagataella pastoris* as described previously *(**25*–*27**)*. A transcription elongation factor, TFIIS, was prepared using a bacterial expression system as described previously *(**27**,*  *28**)*. The transcription reaction on the nucleosome was conducted by mixing the nucleosome (0.1 μM), RNAPII (0.1 μM), TFIIS (0.1 μM) and DY647 fluorescently labeled RNA primer (0.4 μM) (5′-DY647-AUAAUUAGCUC-3′) (Dharmacon) in 13 μl of reaction solution, containing 26 mM HEPES-KOH (pH 7.5), 5 mM MgCl_2_, 50 mM potassium acetate, 0.2 μM zinc acetate, 20 μM Tris(2-carboxyethyl) phosphine, 0.1 mM DTT, 1.5% glycerol, 400 μM UTP, 400 μM CTP, 400 μM GTP and 400 μM ATP, at 4°C. The reaction mixtures were incubated for 0, 3, 6, 9, 12 and 15 min. At each timepoint, an aliquot (2 μl) was taken and mixed with 2 μl of stop solution (50 mM Tris–HCl (pH 8.0), 75 mM EDTA, 0.5 mg/ml proteinase K (Roche) and 4 M urea). The RNA transcripts were denatured by adding Hi-Di formamide (Applied Biosystems) and heating at 95°C for 10 min. The resulting RNA transcripts were analyzed by 10% denaturing polyacrylamide gel electrophoresis (PAGE) in 1× TBE buffer (90 mM Tris base, 90 mM boric acid and 2 mM EDTA). The DY647 fluorescence signals of the elongated RNA products were detected using an Amersham Typhoon Imager (Cytiva). The band intensities of the run-off transcripts were quantitated with the ImageQuant™ TL software (Cytiva).

### Preparation of the H3.8 nucleosome for cryo-EM analysis

The purified H3.8 nucleosome containing the 145-bp Widom 601L DNA was stabilized by the gradient fixation (GraFix) method *(**29**)*, using sucrose and paraformaldehyde. The gradient solution was prepared with low buffer (10 mM HEPES–NaOH (pH 7.5), 20 mM NaCl, 1 mM DTT and 5% sucrose) and high buffer (10 mM HEPES–NaOH (pH 7.5), 20 mM NaCl, 1 mM DTT, 20% sucrose and 4% paraformaldehyde), using a Gradient Master 108 (SK BIO International). The H3.8 nucleosome (0.4 nmol) was applied onto the top of the gradient solution, and fractionated by centrifugation at 27,000 rpm for 16 h at 4°C, using an SW41Ti rotor (Beckman Coulter). After centrifugation, the fractions were analyzed by non-denaturing 6% PAGE with EtBr staining. The fractions containing the nucleosome were collected. The sample buffer was exchanged to final buffer (20 mM HEPES–KOH (pH 7.5), 50 mM potassium acetate, 0.2 μM zinc acetate and 0.1 mM Tris(2-carboxyethyl)phosphine) by a PD-10 column (Cytiva). The nucleosome sample was then concentrated to 10 μM using an Amicon Ultra 30 K centrifugal filter (Merck Millipore). A 2.5-μl portion of the sample was plunge frozen by vitrification, using a Vitrobot Mark IV (Thermo Fisher Scientific) on a Quantifoil R1.2/1.3200-mesh copper grid, which was plasma cleaned for 20 s by a Solarus II (Gatan).

### Cryo-EM data acquisition of the H3.8 nucleosome

Cryo-EM images of the H3.8 nucleosome were collected by a Krios G4 transmission electron microscope (Thermo Fisher Scientific), operating at 300 kV and equipped with a K3 direct electron detector with a Quantum GIF imaging filter (Gatan), using a slit width of 20 eV. The data collection was performed using the EPU software (Thermo Fisher Scientific), with a pixel size of 1.06 Å and defocus values ranging from −1.0 to −2.5 μm. Each micrograph of the H3.8 nucleosome was recorded with a 4.5-s exposure time, and then fractionated into 40 frames with a total dose of 61.2 electrons per Å^2^.

### Image processing

In total, 10,562 micrographs were stacked and motion-corrected using MOTIONCOR2 *(**30**)* with dose weighting. The estimation of contrast transfer function (CTF) from the dose-weighted micrographs was performed using CTFFIND4 *(**31**)*. RELION4 *(**32**)* was used for all subsequent image processing. From 9969 micrographs, 9,365,020 particles were automatically picked, based on the 2D class averages generated from 400 micrographs of the collected data set and extracted with a binning factor of 2 (pixel size of 2.12 Å/pixel). In total, 8,822,886 particles were further selected by 2D classification. The crystal structure of a *Xenopus laevis* nucleosome (PDB ID: 3UT9 *(**33**)*) was used as the initial model for 3D classification. After two rounds of 3D classification, 3,900,336 particles were selected and re-extracted without binning for 3D refinement, followed by Bayesian polishing and CTF refinement. The refined map of the H3.8 nucleosome was sharpened with a B-factor of −73.1 Å^2^. The resolution of the final 3D map was 2.26 Å, as estimated by the gold standard Fourier Shell Correlation (FSC) at FSC = 0.143 *(**34**)*. The local resolution of the map was calculated by RELION4 *(**32**)* and visualized with UCSF ChimeraX (version 1.6.1) *(**35**,*  *36**)*.

**Table 1:** Cryo-EM data collection, image processing, model building and validation statistics

Samples	H3.8 nucleosome (EMD-37070, PDB ID: 8KB5)
**Data collection**	
Electron microscope	Krios G4
Camera	K3
Pixel size (Å/pix)	1.06
Defocus range (μm)	−1.0 to −2.5
Exposure time (s)	4.5
Total dose (e/Å^2^)	61.2
Movie frames (no.)	40
Total micrographs (no.)	10,562
**Reconstruction**	
Software	Relion 4
Particles for 2D classification	9,365,020
Particles for 3D classification	8,822,886
Particles in the final map (no.)	3,900,336
Symmetry	C1
Final resolution (Å)	2.3
FSC threshold	0.143
Map sharpening B factor (Å^2^)	−73.1
**Model building**	
Software	Coot
**Refinement**	
Software	Phenix, Coot, ISOLDE
**Model composition**	
Protein	758
Nucleotide	290
**Validation**	
MolProbity score	0.50
Clash score	0.00
	
R.m.s. deviations	
Bond lengths (Å)	0.007
Bond angles (°)	1.094
**Ramachandran plot**	
Favored (%)	99.33
Allowed (%)	0.67
Outliers (%)	0

**Fig. 1:**
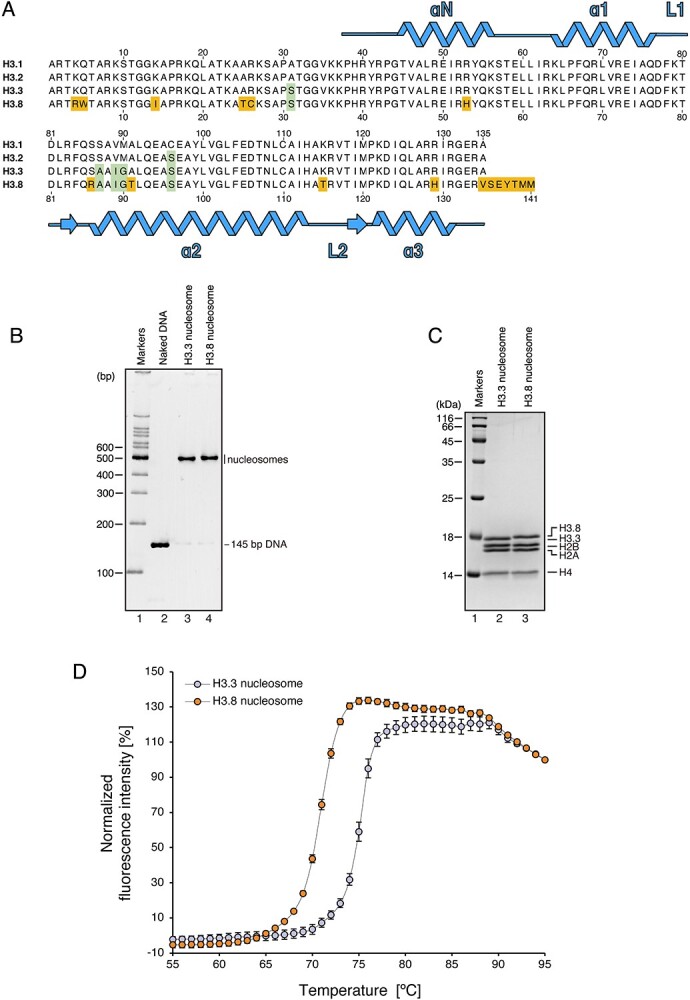
**Structural instability of the nucleosome formed with H3.8.** (A) Amino acid sequence alignment of human histones H3.1, H3.2, H3.3 and H3.8. Conserved residues between H3.3 and H3.8, but not H3.1, are highlighted by green boxes, and H3.8-specific residues are highlighted by orange boxes. (B) Non-denaturing PAGE analysis of purified nucleosomes containing H3.3 or H3.8 with EtBr staining. (C) SDS-PAGE analysis of the purified nucleosomes containing H3.3 or H3.8 with CBB staining. (D) Thermal denaturation curve of the H3.8 nucleosome. Histone proteins thermally dissociated from the nucleosomes are detected by SYPRO Orange fluorescent dye, which hydrophobically binds to the surface of the denatured histones. The error bars indicate standard deviations (n = 3). Orange and blue circles represent experiments with the H3.8 nucleosome and H3.3 nucleosome, respectively.

**Fig. 2:**
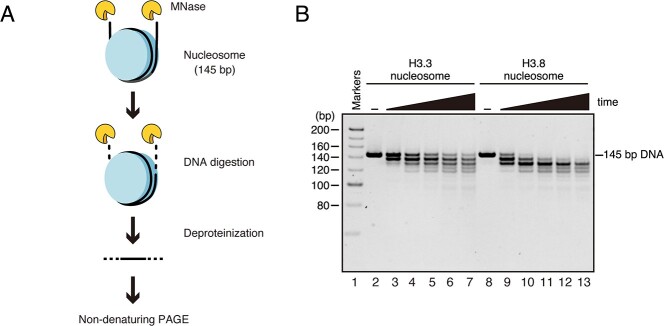
**DNA end flexibility of the H3.8 nucleosome.** (A) Schematic diagram of the micrococcal nuclease (MNase) treatment assay. The DNA ends of the nucleosome are preferentially digested by MNase. After deproteinization, the resulting DNA fragments were analyzed by non-denaturing PAGE. (B) A representative gel image of the MNase treatment assay. The nucleosomes containing H3.3 (lanes 2–7) or H3.8 (lanes 8–13) were incubated in the presence of MNase for 0, 3, 6, 9, 12 and 15 min. The resulting DNA fragments were analyzed by non-denaturing PAGE with EtBr staining. The results were confirmed to be reproducible by two additional independent experiments (shown in Supplementary Fig. S1).

**Fig. 3:**
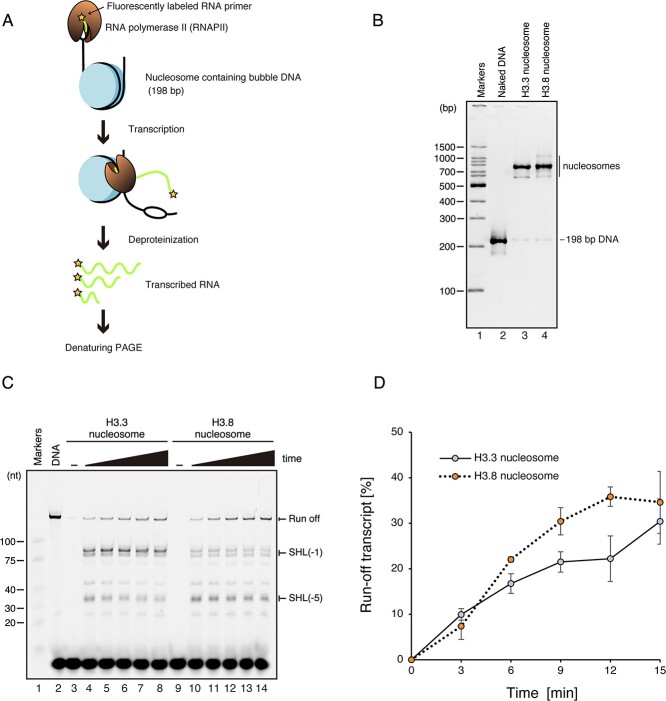
**RNAPII transcription on the H3.8 nucleosome.** (A) Schematic diagram of the RNAPII transcription assay. (B) Non-denaturing PAGE analysis of purified nucleosomes containing a 198-bp DNA for transcription. The gel was stained by EtBr. (C) Representative gel image of the RNAPII transcription assay. The nucleosomes containing H3.3 (lanes 3–8) or H3.8 (lanes 9–14) were incubated in the presence of RNAPII, TFIIS, dNTPs and DY647 fluorescently labeled RNA primer, and the transcription reaction was conducted for 0 min (lanes 3 and 9), 3 min (lanes 4 and 10), 6 min (lanes 5 and 11), 9 min (lanes 6 and 12), 12 min (lanes 7 and 13) and 15 min (lanes 8 and 14). After termination of the transcription reaction, the resulting elongated RNA fragments were fractionated by denaturing PAGE and detected by DY647 fluorescence. The results were confirmed to be reproducible by two additional independent experiments (shown in Supplementary Fig. S2). (D) Graphical representation of the RNAPII transcription assay. The band intensities corresponding to the run-off transcripts in the H3.3 and H3.8 nucleosomes were quantitated, and the run-off transcripts (%) relative to that of the naked DNA template were plotted against the reaction time. The error bars indicate standard deviations (n = 3).

### Model building

The atomic model of the H3.8 nucleosome was built based on the high-resolution cryo-EM structure of the human H3.1 nucleosome containing the Widom 601L DNA (PDB ID: 7VZ4). The atomic coordinates of the H3.1 nucleosome were rigid-body fitted into the density map of the H3.8 nucleosome, using UCSF Chimera *(**37**)*. The amino acid residues of histone H3.1 were replaced with those of histone H3.8 using COOT (version 0.9.8.3) *(**38**)*. The resulting atomic model was refined by phenix.real_space_refine *(**39**,*  *40**)*, followed by manual editing with COOT and interactive MDFF in ISOLDE (ChimeraX version 1.3/ISOLDE version 1.3) *(**41**)*. The final model of the H3.8 nucleosome was evaluated by MolProbity (*(**42**)*; Table 1) in Phenix (version 1.20.1-4487) *(**40**)*. All structural figures were produced with PyMOL (The PyMOL Molecular Graphics System, Version 2.5, Schrödinger, L.L.C.) and ChimeraX (version 1.6.1) *(**35**,*  *36**)*.

**Fig. 4:**
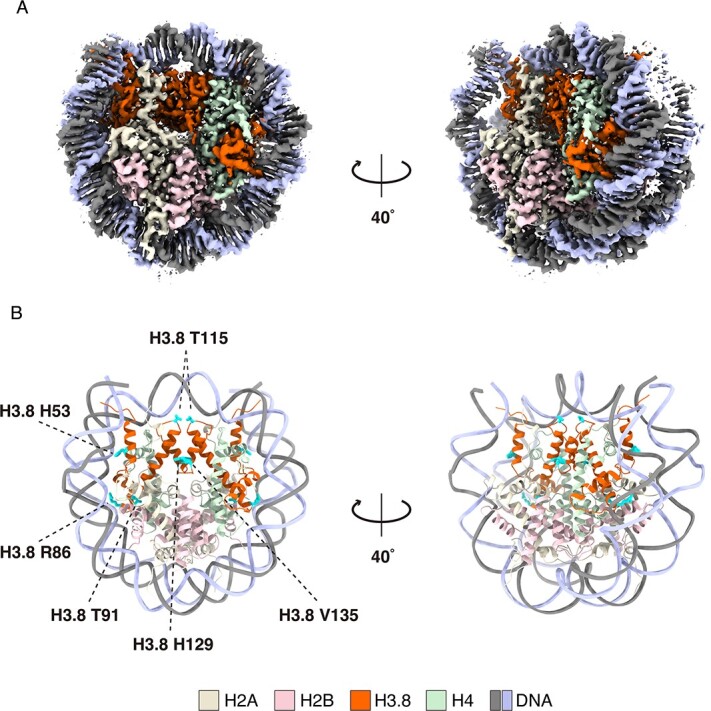
**Cryo-EM structure of the H3.8 nucleosome.** (A) Cryo-EM density map of the H3.8 nucleosome. Histones H3.8, H2A, H2B and H4 are colored orange, yellow, pink and green, respectively. DNA strands are colored gray and blue. (B) Atomic model of the H3.8 nucleosome. The H3.8-specific His53, Arg86, Thr91, Thr115, His129 and Val135 residues are shown with cyan-colored side chains.

**Fig. 5:**
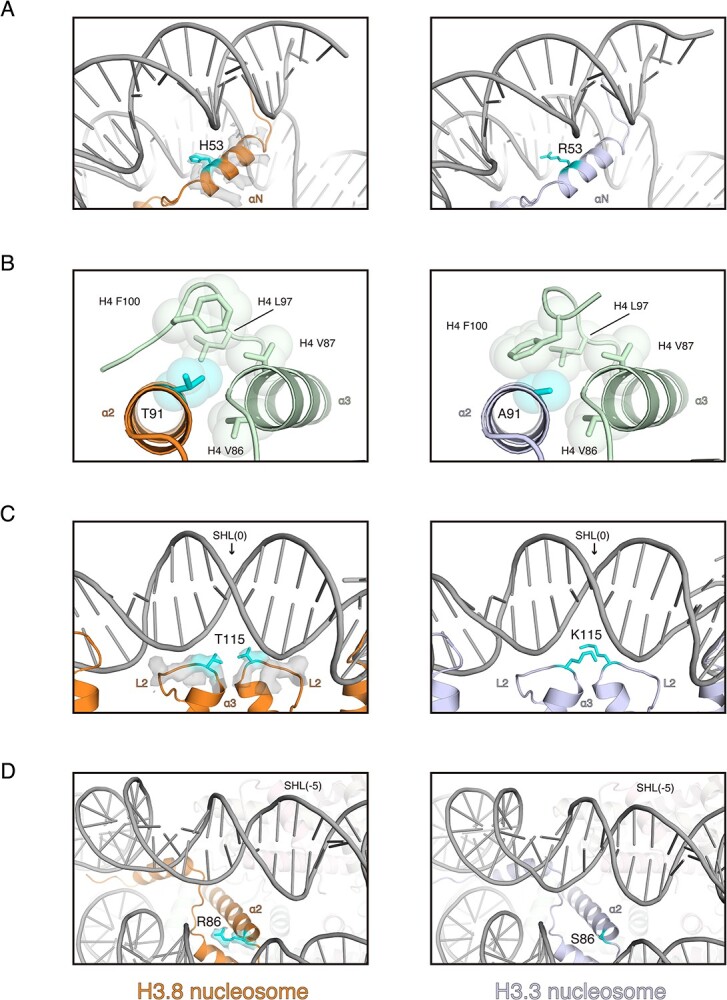
**Structural comparison of the nucleosomes containing H3.3 and H3.8.** (A) Close-up views around the entry/exit DNA regions of the H3.8 nucleosome (left panel) and the H3.3 nucleosome (right panel). The position 53 residues of H3.3 and H3.8 are shown in cyan with side chains. The cryo-EM map of the H3.8 αN helix region is overlaid on the cartoon model. (B) Structural comparison around position 91 in the H3.8 nucleosome (left panel) and the H3.3 nucleosome (right panel). The position 91 residues of H3.3 and H3.8 are shown in cyan with side chains. The van der Waals surfaces of the side chain atoms around the position 91 residues of H3 are represented as spheres. (C) Close-up views around SHL(0) of the H3.8 nucleosome (left panel) and the H3.3 nucleosome (right panel). The position 115 residues of H3.3 and H3.8 are shown in cyan with side chains. The cryo-EM map of the H3.8L2 loop region is overlaid on the cartoon model. (D) Close-up views around SHL(−5) of the H3.8 nucleosome (left panel) and the H3.3 nucleosome (right panel). The position 86 residues of H3.3 and H3.8 are shown in cyan with side chains. The cryo-EM map of the H3.8 Arg86 is overlaid on the cartoon model. The PDB ID of the H3.3 nucleosome structure shown in this figure is 5X7X.

## Results and Discussion

### Reconstitution of the H3.8 nucleosome

H3.8 contains 11 amino acid substitutions and a 6-amino acid extension at the C-terminus, as compared with H3.3 (Fig. 1A). Detailed structural and biochemical analyses of the H3.8 nucleosome have not been performed, because the H3.8 nucleosome reconstituted with the native α-satellite DNA sequence was extremely unstable *(**18**)*. To overcome this problem, we reconstituted the H3.8 nucleosome with a palindromic DNA fragment containing the Widom 601L sequence, which forms stably positioned nucleosomes *in vitro (**33**,*  *43**)*. As shown in Fig. 1B and C, the H3.8 and H3.3 nucleosomes were efficiently reconstituted with the 145-bp palindromic Widom 601L DNA. We then performed the thermal stability assay. Consistent with the previous results obtained with the α-satellite DNA, the H3.8 nucleosome with the Widom 601L DNA was less stable than the H3.3 nucleosome (Fig. 1D). In the H3.8 nucleosome with the Widom 601L DNA, the mid-point of the H2A–H2B dissociation was approximately 4°C higher than that of the H3.8 nucleosome with an α-satellite DNA, as previously reported *(**18**)*.

### Enhanced accessibility of the entry/exit DNA regions of the H3.8 nucleosome

To study the DNA accessibility of the H3.8 nucleosome, we performed a micrococcal nuclease (MNase) treatment assay with the H3.8 and H3.3 nucleosomes and compared the susceptibility of the DNA ends to MNase (Fig. 2A). MNase is an endo/exonuclease that preferentially digests the DNA detached from the histone surface in the nucleosome *(**44**)*. The DNA ends of the H3.8 nucleosome were more susceptible to MNase than those of the H3.3 nucleosome (Fig. 2B and Supplementary Fig. S1). This suggested that the replacement of H3.3 with H3.8 renders the entry/exit DNA regions more accessible and/or flexible.

### Transcription elongation in the H3.8 nucleosome by RNA polymerase II

The central DNA region located at the dyad axis of the nucleosome is termed superhelical location (SHL) 0. We previously reported that RNA polymerase II (RNAPII) transcribes the DNA wrapped in the nucleosome by gradually peeling the nucleosomal DNA up to the SHL(0) position, which is 73 bp ahead from the entry DNA site *(**45**)*. We also found that RNAPII pauses at the SHL(−5) and SHL(−1) positions, which are about 20 and 60 bp from the entry DNA site of the nucleosome, respectively *(**23**,*  *28**)*. The nucleosome is disassembled when RNAPII transcribes beyond the SHL(0) position, and then reassembled behind the transcribing RNAPII *(**45**)*.

To determine how RNAPII transcribes the H3.8 nucleosome, which has different DNA wrapping at its entry/exit regions, we reconstituted nucleosomes containing H3.8 or H3.3 with the modified Widom 601 DNA sequence *(**23**)*. A linker DNA containing a mismatched bubble region (9 bases) was ligated to one end of the reconstituted nucleosome to serve as the RNAPII loading site for transcription (Fig. 3A). The H3.8 and H3.3 nucleosomes reconstituted for the RNAPII transcription were highly purified and contained only a trace amount of naked DNA (Fig. 3B). The nucleosome containing H3.8 or H3.3 was then transcribed by RNAPII in the presence of TFIIS. In the H3.8 nucleosome, the amount of the run-off transcript was slightly increased as compared to the H3.3 nucleosome (Fig. 3C, D and Supplementary Fig. S2). We found that the RNAPII pausing at the SHL(−5) and SHL(−1) positions is conserved in the H3.8 nucleosome (Fig. 3C and Supplementary Fig. S2). In the H3.8 nucleosome, the amount of RNA product corresponding to RNAPII pausing around the SHL(−1) position was drastically decreased as compared to the H3.3 nucleosome (Fig. 3C and Supplementary Fig. S2). On the other hand, the amount of RNA product corresponding to the RNAPII pausing around the SHL(−5) position was substantially increased (Fig. 3C and Supplementary Fig. S2). Therefore, in the H3.8 nucleosome, RNAPII transcribes the DNA around the SHL(−1) position more efficiently than in the H3.3 nucleosome, and the RNAPII pausing around the SHL(−5) position is enhanced.

### Cryo-EM structure of the H3.8 nucleosome

We next performed the cryo-electron microscope (cryo-EM) analysis of the H3.8 nucleosome with the 145-bp palindromic Widom 601L DNA. The reconstituted H3.8 nucleosome was prepared by sucrose gradient ultracentrifugation in the presence of glutaraldehyde (GraFix). The cryo-EM structure of the H3.8 nucleosome was determined at 2.3 Å resolution (Fig. 4A, B and Supplementary Fig. S3A-E).

Under tension force, the nucleosomal DNA ends are asymmetrically unwrapped *(**46**)*. The asymmetric DNA wrapping is also observed in the *Giardia lamblia* nucleosome with a palindromic Widom 601L DNA *(**47**)*. We found that the entry/exit DNA regions are asymmetrically wrapped in the H3.8 nucleosome structure (Fig. 4A and Supplementary Fig. S3B). The H3.8-specific His53 residue, corresponding to the H3.3 Arg53 residue, is located near the entry/exit DNA regions (Fig. 5A). Arg has a basic side chain and is the main amino acid residue that directly binds to the DNA backbone in the nucleosome *(**1**,*  *5**)*. Therefore, the Arg–His substitution in H3.8 may reduce the histone–DNA interactions around the entry/exit DNA region of the nucleosome and, thus, render the DNA more accessible. Substitutions at position 53 are also observed in the CENP-A and mouse H3mm18 H3 variants. Interestingly, like the H3.8 nucleosome, the nucleosomes containing CENP-A and H3mm18 also exhibited enhanced DNA end accessibility *(**11**,*  *48**,*  *49**)*. The 53rd residue of H3 variants may be important for providing the diversity of the DNA dynamics in the nucleosome.

The H3.3 Ala91 residue is located in a hydrophobic pocket with the H4 Val86, Val87, Leu97 and Phe100 residues (Fig. 5B). Interestingly, in H3.8, the 91st residue is a hydrophilic Thr residue, which drastically changes the orientation of the H4 Phe100 side chain in the H3.8 nucleosome (Fig. 5B). This Ala–Thr substitution in H3.8 may weaken the hydrophobic interaction between H3 and H4 and reduce the stability of the H3.8 nucleosome.

We found that the H3.8-specific Thr115 residue, corresponding to the H3.3 Lys115 residue, is located close to the DNA backbone around the SHL(0) position of the nucleosome (Fig. 5C). Thr is a neutral residue and does not form electrostatic interactions with the DNA backbone. Therefore, the Lys–Thr substitution in H3.8 may reduce the histone–DNA interactions around the SHL(0) position and facilitate the alleviation of RNAPII pausing around SHL(−1).

In the H3.8 nucleosome structure, the H3.8-specific Arg86 residue, corresponding to the H3.3 Ser86 residue, is located near the DNA backbone around the SHL(−5) region (Fig. 5D). The Arg residue may bind to the DNA backbone and may stabilize the local histone–DNA contact. This H3.8-specific Arg86 residue may explain the mechanism by which the RNAPII pausing around the SHL(−5) position is enhanced by the addition local histone–DNA interaction in the H3.8 nucleosome.

## Conclusion

In the present study, we successfully reconstituted the nucleosome containing the human histone H3.8 variant. In comparison with the H3.3 nucleosome, we found that (i) the H3.8 nucleosome is less stable, (ii) the entry/exit DNA regions of the H3.8 nucleosome are more accessible, (iii) the RNAPII pausing around the SHL(−1) position of the nucleosome is alleviated and (iv) the RNAPII pausing around the SHL(−5) position of the nucleosome is enhanced. These biochemical characteristics may be explained by the H3.8-specific amino acid residues, such as His53, Thr91, Thr115 and Arg86, which are located in the contact surfaces with the entry/exit DNA regions, a hydrophobic core with H4, the SHL(0) DNA region and the SHL(−5) region, respectively, as revealed by the cryo-EM structure of the H3.8 nucleosome. These biochemical and structural features of the H3.8 nucleosome form a basis to elucidate its biological relevance and provide new insights into understanding the roles of histone variants in cells.

## Supplementary Material

Web_Material_mvad069

## References

[1] Luger, K., Mäder, A.W., Richmond, R.K., Sargent, D.F., and Richmond, T.J. (1997) Crystal structure of the nucleosome core particle at 2.8 Å resolution. Nature 389, 251–260 9305837 10.1038/38444

[2] Talbert, P.B. and Henikoff, S. (2017) Histone variants on the move: substrates for chromatin dynamics. Nat. Rev. Mol. Cell Biol. 18, 115–126 27924075 10.1038/nrm.2016.148

[3] Tagami, H., Ray-Gallet, D., Almouzni, G., and Nakatani, Y. (2004) Histone H3.1 and H3.3 complexes mediate nucleosome assembly pathways dependent or independent of DNA synthesis. Cell 116, 51–61 14718166 10.1016/s0092-8674(03)01064-x

[4] Talbert, P.B., Ahmad, K., Almouzni, G., Ausió, J., Berger, F., Bhalla, P.L., Bonner, W.M., Cande, W., Chadwick, B.P., Chan, S.W.L., Cross, G.A., Cui, L., Dimitrov, S.I., Doenecke, D., Eirin-López, J.M., Gorovsky, M.A., Hake, S.B., Hamkalo, B.A., Holec, S., Jacobsen, S.E., Kamieniarz, K., Khochbin, S., Ladurner, A.G., Landsman, D., Latham, J.A., Loppin, B., Malik, H.S., Marzluff, W.F., Pehrson, J.R., Postberg, J., Schneider, R., Singh, M.B., Smith, M.M., Thompson, E., Torres-Padilla, M.E., Tremethick, D.J., Turner, B.M., Waterborg, J.H., Wollmann, H., Yelagandula, R., Zhu, B., and Henikoff, S. (2012) A unified phylogeny-based nomenclature for histone variants. Epigenetics Chromatin 5, 7 22650316 10.1186/1756-8935-5-7 PMC3380720

[5] Koyama, M. and Kurumizaka, H. (2018) Structural diversity of the nucleosome. J. Biochem. 163, 85–95 29161414 10.1093/jb/mvx081

[6] Kurumizaka, H., Horikoshi, N., Tachiwana, H., and Kagawa, W. (2013) Current progress on structural studies of nucleosomes containing histone H3 variants. Curr. Opin. Struct. Biol. 23, 109–115 23265997 10.1016/j.sbi.2012.10.009

[7] Kurumizaka, H., Kujirai, T., and Takizawa, Y. (2021) Contributions of histone variants in nucleosome structure and function. J. Mol. Biol. 433, 166678 33065110 10.1016/j.jmb.2020.10.012

[8] Takizawa, Y. and Kurumizaka, H. (2022) Chromatin structure meets cryo-EM: dynamic building blocks of the functional architecture. Biochim. Biophys. Acta Gene Regul. Mech. 1865, 194851 35952957 10.1016/j.bbagrm.2022.194851

[9] Conde e Silva, N., Black, B.E., Sivolob, A., Filipski, J., Cleveland, D.W., and Prunell, A. (2007) CENP-A-containing nucleosomes: easier disassembly versus exclusive centromeric localization. J. Mol. Biol. 370, 555–573 17524417 10.1016/j.jmb.2007.04.064

[10] Dechassa, M.L., Wyns, K., Li, M., Hall, M.A., Wang, M.D., and Luger, K. (2011) Structure and Scm 3-mediated assembly of budding yeast centromeric nucleosomes. Nat. Commun. 2, 313 21587230 10.1038/ncomms1320 PMC3112535

[11] Tachiwana, H., Kagawa, W., Shiga, T., Osakabe, A., Miya, Y., Saito, K., Hayashi-Takanaka, Y., Oda, T., Sato, M., Park, S.Y., Kimura, H., and Kurumizaka, H. (2011) Crystal structure of the human centromeric nucleosome containing CENP-A. Nature 476, 232–235 21743476 10.1038/nature10258

[12] Panchenko, T., Sorensen, T.C., Woodcock, C.L., Kan, Z.Y., Wood, S., Resch, M.G., Luger, K., Englander, S.W., Hansen, J.C., and Black, B.E. (2011) Replacement of histone H3 with CENP-A directs global nucleosome array condensation and loosening of nucleosome superhelical termini. Proc. Natl. Acad. Sci. U. S. A. 108, 16588–16593 21949362 10.1073/pnas.1113621108 PMC3189058

[13] Kujirai, T., Horikoshi, N., Sato, K., Maehara, K., Machida, S., Osakabe, A., Kimura, H., Ohkawa, Y., and Kurumizaka, H. (2016) Structure and function of human histone H3.Y nucleosome. Nucleic Acids Res. 44, 6127–6141 27016736 10.1093/nar/gkw202 PMC5291245

[14] Ueda, J., Harada, A., Urahama, T., Machida, S., Maehara, K., Hada, M., Makino, Y., Nogami, J., Horikoshi, N., Osakabe, A., Taguchi, H., Tanaka, H., Tachiwana, H., Yao, T., Yamada, M., Iwamoto, T., Isotani, A., Ikawa, M., Tachibana, T., Okada, Y., Kimura, H., Ohkawa, Y., Kurumizaka, H., and Yamagata, K. (2017) Testis-specific histone variant H3t gene is essential for entry into spermatogenesis. Cell Rep. 18, 593–600 28099840 10.1016/j.celrep.2016.12.065

[15] Tachiwana, H., Kagawa, W., Osakabe, A., Kawaguchi, K., Shiga, T., Hayashi-Takanaka, Y., Kimura, H., and Kurumizaka, H. (2010) Structural basis of instability of the nucleosome containing a testis-specific histone variant, human H3T. Proc. Natl. Acad. Sci. U. S. A. 107, 10454–10459 20498094 10.1073/pnas.1003064107 PMC2890842

[16] Urahama, T., Harada, A., Maehara, K., Horikoshi, N., Sato, K., Sato, Y., Shiraishi, K., Sugino, N., Osakabe, A., Tachiwana, H., Kagawa, W., Kimura, H., Ohkawa, Y., and Kurumizaka, H. (2016) Histone H3.5 forms an unstable nucleosome and accumulates around transcription start sites in human testis. Epigenetics Chromatin 9, 2 26779285 10.1186/s13072-016-0051-y PMC4714512

[17] Maehara, K., Harada, A., Sato, Y., Matsumoto, M., Nakayama, K.I., Kimura, H., and Ohkawa, Y. (2015) Tissue-specific expression of histone H3 variants diversified after species separation. Epigenetics Chromatin 8, 1–17 26388943 10.1186/s13072-015-0027-3 PMC4574566

[18] Taguchi, H., Xie, Y., Horikoshi, N., Maehara, K., Harada, A., Nogami, J., Sato, K., Arimura, Y., Osakabe, A., Kujirai, T., Iwasaki, T., Semba, Y., Tachibana, T., Kimura, H., Ohkawa, Y., and Kurumizaka, H. (2017) Crystal structure and characterization of novel human histone H3 variants, H3.6, H3.7, and H3.8. Biochemistry 56, 2184–2196 28374988 10.1021/acs.biochem.6b01098

[19] Ricketts, M.D., Frederick, B., Hoff, H., Tang, Y., Schultz, D.C., Rai, T.S., Vizioli, M.G., Adams, P.D., and Marmorstein, R. (2015) Ubinuclein-1 confers histone H3.3-specific-binding by the HIRA histone chaperone complex. Nat. Commun. 6, 1–11 10.1038/ncomms8711 PMC4510971 26159857

[20] Elsässer, S.J., Huang, H., Lewis, P.W., Chin, J.W., Allis, C.D., and Patel, D.J. (2012) DAXX envelops a histone H3.3-H4 dimer for H3.3-specific recognition. Nature 491, 560–565 23075851 10.1038/nature11608 PMC4056191

[21] Liu, C.P., Xiong, C., Wang, M., Yu, Z., Yang, N., Chen, P., Zhang, Z., Li, G., and Xu, R.M. (2012) Structure of the variant histone H3.3–H4 heterodimer in complex with its chaperone DAXX. Nat. Struct. Mol. Biol. 19, 1287–1292 23142979 10.1038/nsmb.2439 PMC3932182

[22] Kujirai, T., Arimura, Y., Fujita, R., Horikoshi, N., Machida, S., and Kurumizaka, H. (2018) Methods for preparing nucleosomes containing histone variants. Methods Mol. Biol. 1832, 3–20 30073519 10.1007/978-1-4939-8663-7_1

[23] Kujirai, T., Ehara, H., Fujino, Y., Shirouzu, M., Sekine, S.I., and Kurumizaka, H. (2018) Structural basis of the nucleosome transition during RNA polymerase II passage. Science 362, 595–598 30287617 10.1126/science.aau9904

[24] Taguchi, H., Horikoshi, N., Arimura, Y., and Kurumizaka, H. (2014) A method for evaluating nucleosome stability with a protein-binding fluorescent dye. Methods 70, 119–126 25220913 10.1016/j.ymeth.2014.08.019

[25] Higo, T., Suka, N., Ehara, H., Wakamori, M., Sato, S., Maeda, H., Sekine, S.I., Umehara, T., and Yokoyama, S. (2014) Development of a hexahistidine-3× FLAG-tandem affinity purification method for endogenous protein complexes in *Pichia pastoris*. J. Struct. Funct. Genom. 15, 191–199 10.1007/s10969-014-9190-1 PMC4237914 25398586

[26] Ehara, H., Umehara, T., Sekine, S.I., and Yokoyama, S. (2017) Crystal structure of RNA polymerase II from *Komagataella pastoris*. Biochem. Biophys. Res. Commun. 487, 230–235 28412353 10.1016/j.bbrc.2017.04.039

[27] Ehara, H., Yokoyama, T., Shigematsu, H., Yokoyama, S., Shirouzu, M., and Sekine, S.I. (2017) Structure of the complete elongation complex of RNA polymerase II with basal factors. Science 357, 921–924 28775211 10.1126/science.aan8552

[28] Ehara, H., Kujirai, T., Fujino, Y., Shirouzu, M., Kurumizaka, H., and Sekine, S.I. (2019) Structural insight into nucleosome transcription by RNA polymerase II with elongation factors. Science 363, 744–747 30733384 10.1126/science.aav8912

[29] Stark, H. (2010) GraFix: stabilization of fragile macromolecular complexes for single particle Cryo-EM. Methods Enzymol. 481, 109–126 20887855 10.1016/S0076-6879(10)81005-5

[30] Zheng, S.Q., Palovcak, E., Armache, J.P., Verba, K.A., Cheng, Y., and Agard, D.A. (2017) MotionCor 2: anisotropic correction of beam-induced motion for improved cryo-electron microscopy. Nat. Methods 14, 331–332 28250466 10.1038/nmeth.4193 PMC5494038

[31] Rohou, A. and Grigorieff, N. (2015) CTFFIND4: fast and accurate defocus estimation from electron micrographs. J. Struct. Biol. 192, 216–221 26278980 10.1016/j.jsb.2015.08.008 PMC6760662

[32] Kimanius, D., Dong, L., Sharov, G., Nakane, T., and Scheres, S.H.W. (2021) New tools for automated cryo-EM single-particle analysis in RELION-4.0. Biochem. J. 478, 4169–4185 34783343 10.1042/BCJ20210708 PMC8786306

[33] Chua, E.Y.D., Vasudevan, D., Davey, G.E., Wu, B., and Davey, C.A. (2012) The mechanics behind DNA sequence-dependent properties of the nucleosome. Nucleic Acids Res. 40, 6338–6352 22453276 10.1093/nar/gks261 PMC3401446

[34] Scheres, S.H.W. (2016) Processing of structurally heterogeneous cryo-EM data in RELION. Methods Enzymol. 579, 125–157 27572726 10.1016/bs.mie.2016.04.012

[35] Goddard, T.D., Huang, C.C., Meng, E.C., Pettersen, E.F., Couch, G.S., Morris, J.H., and Ferrin, T.E. (2018) UCSF ChimeraX: meeting modern challenges in visualization and analysis. Protein Sci. 27, 14–25 28710774 10.1002/pro.3235 PMC5734306

[36] Pettersen, E.F., Goddard, T.D., Huang, C.C., Meng, E.C., Couch, G.S., Croll, T.I., Morris, J.H., and Ferrin, T.E. (2021) UCSF ChimeraX: structure visualization for researchers, educators, and developers. Protein Sci. 30, 70–82 32881101 10.1002/pro.3943 PMC7737788

[37] Pettersen, E.F., Goddard, T.D., Huang, C.C., Couch, G.S., Greenblatt, D.M., Meng, E.C., and Ferrin, T.E. (2004) UCSF chimera—a visualization system for exploratory research and analysis. J. Comput. Chem. 25, 1605–1612 15264254 10.1002/jcc.20084

[38] Emsley, P., Lohkamp, B., Scott, W.G., and Cowtan, K. (2010) Features and development of Coot. Acta Crystallogr. Sect. D Biol. Crystallogr. 66, 486–501 20383002 10.1107/S0907444910007493 PMC2852313

[39] Afonine, P.V., Poon, B.K., Read, R.J., Sobolev, O.V., Terwilliger, T.C., Urzhumtsev, A., and Adams, P.D. (2018) Real-space refinement in PHENIX for cryo-EM and crystallography. Acta Crystallogr. Sect. D Struct. Biol. 74, 531–544 29872004 10.1107/S2059798318006551 PMC6096492

[40] Liebschner, D., Afonine, P.V., Baker, M.L., Bunkoczi, G., Chen, V.B., Croll, T.I., Hintze, B., Hung, L.W., Jain, S., McCoy, A.J., Moriarty, N.W., Oeffner, R.D., Poon, B.K., Prisant, M.G., Read, R.J., Richardson, J.S., Richardson, D.C., Sammito, M.D., Sobolev, O.V., Stockwell, D.H., Terwilliger, T.C., Urzhumtsev, A.G., Videau, L.L., Williams, C.J., and Adams, P.D. (2019) Macromolecular structure determination using X-rays, neutrons and electrons: recent developments in Phenix. Acta Crystallogr. Sect. D Struct. Biol. 75, 861–877 31588918 10.1107/S2059798319011471 PMC6778852

[41] Croll, T.I. (2018) ISOLDE: a physically realistic environment for model building into low-resolution electron-density maps. Acta Crystallogr. Sect. D Struct. Biol. 74, 519–530 29872003 10.1107/S2059798318002425 PMC6096486

[42] Williams, C.J., Headd, J.J., Moriarty, N.W., Prisant, M.G., Videau, L.L., Deis, L.N., Verma, V., Keedy, D.A., Hintze, B.J., Chen, V.B., Jain, S., Lewis, S.M., Arendall, W.B., 3rd., Snoeyink, J., Adams, P.D., Lovell, S.C., Richardson, J.S., and Richardson, D.C. (2018) MolProbity: more and better reference data for improved all-atom structure validation. Protein Sci. 27, 293–315 29067766 10.1002/pro.3330 PMC5734394

[43] Lowary, P.T. and Widom, J. (1998) New DNA sequence rules for high affinity binding to histone octamer and sequence-directed nucleosome positioning. J. Mol. Biol. 276, 19–42 9514715 10.1006/jmbi.1997.1494

[44] Noll, M., Thomas, J.O., and Kornberg, R.D. (1975) Preparation of native chromatin and damage caused by shearing. Science 187, 1203–1206 17754290 10.1126/science.187.4182.1203

[45] Ehara, H., Kujirai, T., Shirouzu, M., Kurumizaka, H., and Sekine, S.I. (2022) Structural basis of nucleosome disassembly and reassembly by RNAPII elongation complex with FACT. Science 377, eabp9466 35981082 10.1126/science.abp9466

[46] Ngo, T.T., Zhang, Q., Zhou, R., Yodh, J.G., and Ha, T. (2015) Asymmetric unwrapping of nucleosomes under tension directed by DNA local flexibility. Cell 160, 1135–1144 25768909 10.1016/j.cell.2015.02.001 PMC4409768

[47] Sato, S., Takizawa, Y., Hoshikawa, F., Dacher, M., Tanaka, H., Tachiwana, H., Kujirai, T., Iikura, Y., Ho, C.H., Adachi, N., Patwal, I., Flaus, A., and Kurumizaka, H. (2021) Cryo-EM structure of the nucleosome core particle containing *Giardia lamblia* histones. Nucleic Acids Res. 49, 8934–8946 34352093 10.1093/nar/gkab644 PMC8421212

[48] Kono, H., Shirayama, K., Arimura, Y., Tachiwana, H., and Kurumizaka, H. (2015) Two arginine residues suppress the flexibility of nucleosomal DNA in the canonical nucleosome core. PLoS One 10, e0120635 25786215 10.1371/journal.pone.0120635 PMC4365049

[49] Hirai, S., Tomimatsu, K., Miyawaki-Kuwakado, A., Takizawa, Y., Komatsu, T., Tachibana, T., Fukushima, Y., Takeda, Y., Negishi, L., Kujirai, T., Koyama, M., Ohkawa, Y., and Kurumizaka, H. (2022) Unusual nucleosome formation and transcriptome influence by the histone H3mm18 variant. Nucleic Acids Res. 50, 72–91 34929737 10.1093/nar/gkab1137 PMC8855299

