# Supplementary material for: Cryo-EM and biochemical analyses of the nucleosome containing the human histone H3 variant H3.8
Source: J Biochem. 2023 Sep 26;174(6):549–59. doi: 10.1093/jb/mvad069 (PMC10914216; doi:10.1093/jb/mvad069)
Supplement: Web_Material_mvad069 [file web_material_mvad069.zip › Supplementary_figures230919_proof.pdf]

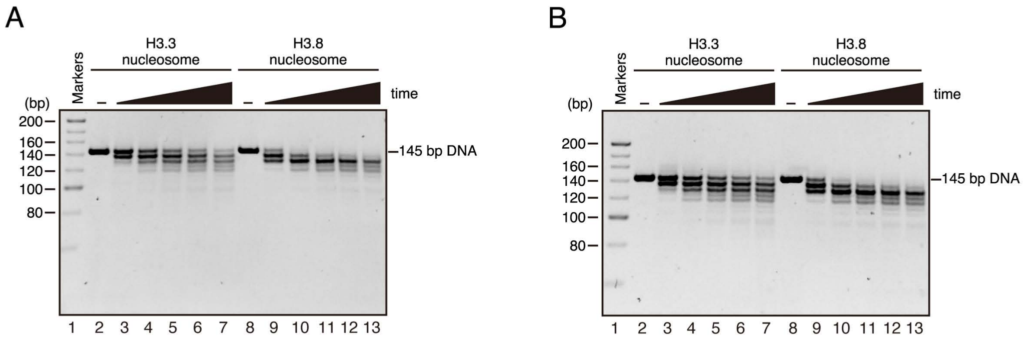

Supplementary Figure S1. (A), (B) Repeated experiments for the MNase treatment assays, as shown in Figure 2B.

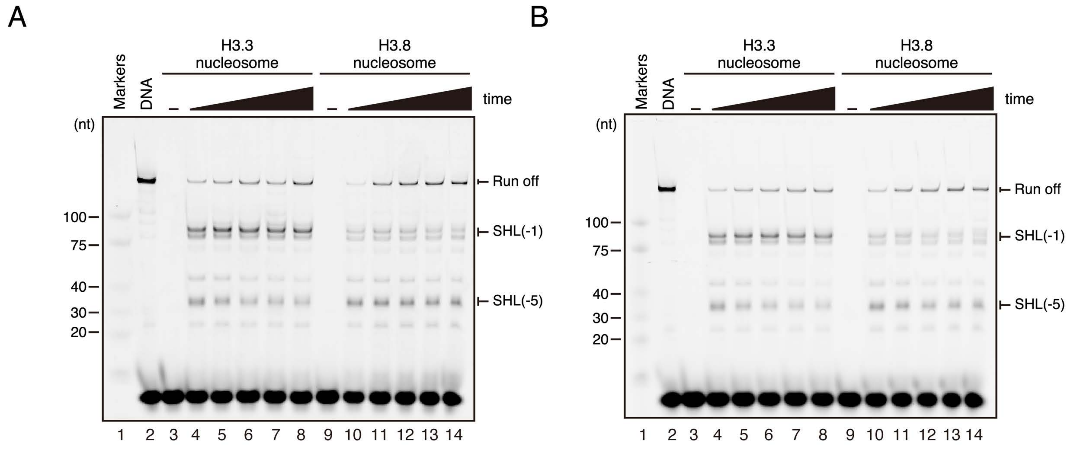

Supplementary Figure S2. (A), (B) Repeated experiments for the RNAPII transcription assays, as shown in Figure 3C.

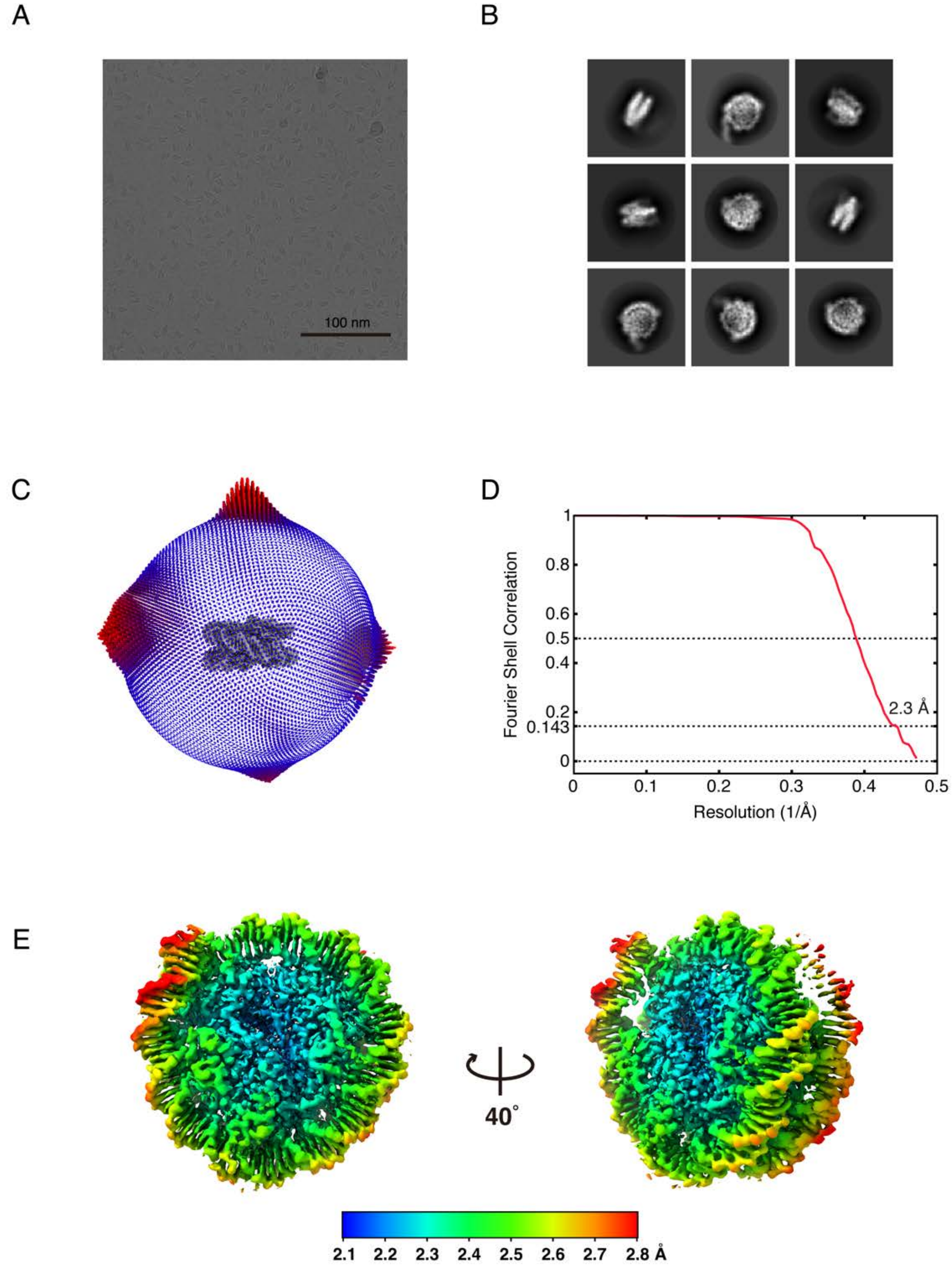

Supplementary Figure S3. Cryo-EM analysis of the H3.8 nucleosome. (A) Representative micrograph of the H3.8 nucleosome. (B) Representative images of 2D class averages of the H3.8 nucleosome structure. (C) Euler angular distribution of the H3.8 nucleosome structure. (D) Fourier Shell Correlation (FSC) curve of the H3.8 nucleosome structure. The resolution of the H3.8 nucleosome structure was estimated to be 2.3 Å by an FSC = 0.143. (E) Local resolution map of the H3.8 nucleosome structure.
